# Madras motor neuron disease (MMND) is distinct from the riboflavin transporter genetic defects that cause Brown–Vialetto–Van Laere syndrome

**DOI:** 10.1016/j.jns.2013.08.003

**Published:** 2013-11-15

**Authors:** Atchayaram Nalini, Amelie Pandraud, Kin Mok, Henry Houlden

**Affiliations:** a Department of Neurology, National Institute of Mental Health and Neurosciences, Bangalore, India; b Department of Molecular Neuroscience, UCL Institute of Neurology, Queen Square, London WC1N 3BG, United Kingdom; c The MRC Centre for Neuromuscular Diseases, UCL Institute of Neurology, Queen Square, London WC1N 3BG, United Kingdom

**Keywords:** Madras motor neuron disease, MMND, Riboflavin receptors, C9ORF72, Brown–Vialetto–Van Laere syndrome, BVVL

## Abstract

**Introduction:**

Madras motor neuron disease (MMND), MMND variant (MMNDV) and Familial MMND (FMMND) have a unique geographic distribution predominantly reported from Southern India. The characteristic features are onset in young, weakness and wasting of limbs, multiple lower cranial nerve palsies and sensorineural hearing loss. There is a considerable overlap in the phenotype of MMND with Brown–Vialetto–Van Laere syndrome (BVVL) Boltshauser syndrome, Nathalie syndrome and Fazio–Londe syndrome. Recently a number of BVVL cases and families have been described with mutations in two riboflavin transporter genes SLC52A2 and SLC52A3 (solute carrier family 52, riboflavin transporter, member 2 and 3 respectively).

**Methods and results:**

We describe six families and four sporadic MMND cases that have been clinically characterized in detail with history, examination, imaging and electrophysiological investigations. We sequenced the SLC52A1, SLC52A2 and SLC52A3 in affected probands and sporadic individuals from the MMND series as well as the C9ORF72 expansion. No genetic defects were identified and the C9ORF72 repeats were all less than 10.

**Conclusions:**

These data suggest that MMND is a distinct clinical subgroup of childhood onset MND patients where the known genetic defects are so far negative. The clinico-genetic features of MMND in comparison with the BVVL group of childhood motor neuron diseases suggest that these diseases are likely to share a common defective biological pathway that may be a combination of genetic and environmental factors.

## 1 Introduction

Madras motor neuron disease (MMND) a unique disorder which was described in 1970, was a name given by Meenakshisundaram et al. to a group of patients from Madras (now called Chennai) located in Southern India [1]. The disease manifests in young individuals with clinical features of thin habitus, wasting and weakness predominantly of distal muscles of the limbs, involvement of facial and bulbar muscles, pyramidal dysfunction and associated sensorineural hearing impairment. The disease was described as a sporadic disorder with benign course [1,2]. Later they described the same disorder in additional 29 cases [2,3]. Subsequently a few more reports from Bangalore and Vellore in the South, Mumbai in the West and Kolkata in the East were published [4–10]. Apart from reports mainly from India three independent cases, one each from Thailand, Italy and China have been reported and curiously the patient in the Italian report had originated from South India [11–13]. Although the earliest reports stated that it was a sporadic benign disorder, we have reported on the familial form of MMND and also observed that the disorders could have a more rapid and fatal course [14]. We have reported on the entity of MMND variant wherein these patients in addition have features of optic atrophy [15].

MMND overlaps clinically with Brown–Vialetto–Van Laere Syndrome (BVVL) [16,17], which in itself is likely allelic to a number of other complex childhood motor neuron diseases such as Boltshauser syndrome [18], Nathalie syndrome [19] and Fazio–Londe syndrome [20]. Recently, mutations in the gene *SLC52A3* (solute carrier family 52, riboflavin transporter, member 3) were identified as a cause of BVVL in a consanguineous family with multiple affected individuals [21,22]. Mutations were subsequently found in 6 additional families. In humans there are 3 members of the riboflavin transporter family, SLC52A1, SLC52A2 and SLC52A3 [23,24]. SLC52A2 and SLC52A3 cause typical BVVL and SLC52A1 causes a complex childhood syndrome with similar neurological features. The great importance of identifying mutations in this patient group is that riboflavin supplementation improves the clinical phenotype.

Here we describe the detailed clinical phenotype and genetic screening of six MMND families and four sporadic cases. The SLC52A1, SLC52A2 and SLC52A3 genes were sequenced and the series was examined for C9ORF72 expansions which have recently been shown to be a common cause of MND [25,26].

## 2 Materials and method

Ten patients with classical features of MMND were studied in this report. All these patients had volunteered and gave written informed consent to perform the genetic analysis for presence of BVVL gene in their blood samples. All patients were evaluated at the National Institute of Mental Health and Neurosciences, a tertiary national referral center for neurological disorders. The medical records were scrutinized and all data regarding the clinical features including electromyography, nerve conduction studies, audiological investigations, visual evoked potentials, CT scan and MRI brain findings were entered into a proforma. After the consent 10 ml of blood for genomic DNA was collected and DNA separated and stored at the Molecular genetics laboratory DNA Bank at the Institute. Patients were informed about the non-availability of facilities in India to genetically diagnose these rare orphan disorders and hence they opted to have the genetic testing for MMND and BVVL syndrome in the UK and the DNA samples were transferred by the patients.

### 2.1 Sanger sequencing

*SLC52A1*, *SLC52A2* and *SLC52A3* PCR primers were designed using Exon Primer so that PCR products would span whole exons and approximately 35 bp of flanking introns (http://ihg.gsf.de/ihg/ExonPrimer.html) (Table 2). PCR primers were also designed for *SLC52A1* and *SLC52A3* as above and previously described [27,28]. PCR amplification was performed using 25 ng of genomic DNA, 12 μl 2× PCR Master Mix (Roche, Indianapolis, IN), 1 μl each of 10 μM forward and reverse  primers, and 2 μl H_2_O. Touchdown PCR was performed as follows: 95 °C for 5 min, followed by 8 cycles of denaturing at 95 °C for 20 s, annealing at 60 °C for 20 s, and extending at 72 °C for 30 s. Following these 8 cycles, 20 cycles of the same conditions were run, however the annealing temperature was decreased by 0.5 °C per cycle until the final annealing temperature reached 50 °C. Finally, 12 cycles were run with denaturing at 95 °C for 20 s, annealing at 50 °C for 20 s, and extending at 72 °C for 30 s. Product underwent final extension at 72 °C for 5 min. PCR amplification products were cleaned with AMPure SPRI beads as per the manufacturer's protocol (Agencourt, Danvers, MA, US).

Approximately 25 ng (roughly 1 μl) of each PCR product was used as template per each sequencing reaction. Sequencing reactions contained 5 × reaction buffer (2 μl), big dye (0.5 μl) (Applied Biosystems, Foster City, CA) and H_2_O (5.5 μl), and the forward or reverse primer that was used for amplification of the original product (1 μl). Conditions were as follows: 25 cycles of denaturation at 95 °C, annealing at 50 °C, and extension at 60 °C were performed. Sequencing reactions were cleaned using CleanSEQ SPRI beads as per the manufacturer's protocol (Agencourt). Sequencing was performed using the 3730 DNA Analyzer (Applied Biosystems).

We screened the GGGGCC expansion in the MMND cohort and the controls using the reversed-prime PCR protocol as previously reported. Briefly, 100 ng of genomic DNA was amplified with a final volume of 20 μl containing 10 μl FastStart PCR Master Mix (Roche), 0.18 mM 7-deaza dGTP, 1 × Qiagen Q solution, 10% DMSO, 0.7 μM reverse primer consisting of four GGGGCC repeats and an anchor tail (TACGCATCCCAGTTTGAGACGGGGGCCGGGGCCGGGGCCGGGG), 1.4 μM 6FAM fluorescent-primer located 280 bp 3′ prime to the repeat sequence (AGTCGCTAGAGGCGAAAGC) and 1.4 μM anchor primer corresponding to the anchor tail of the reverse primer (TACGCATCCCAGTTTGAGACG). A touchdown PCR cycling protocol was used where the initial annealing temperature was lowered from 70 °C to 56 °C in 2 °C increments and a 3 min extension time for each cycle. Fragment length analysis was performed on an ABI 3730XL genetic analyzer (Applied Biosystems Inc., Foster City, CA, USA), and data analyzed using ABI GeneScan software. Each case was analyzed in duplicate to confirm allele sizes.

## 3 Audiological assessment

Hearing acuity was established at octave frequencies from 250 Hz to 8000 Hz for air conduction and 250 Hz to 4000 Hz for bone conduction. Degree of hearing impairment was classified based on the average of air conduction pure tone thresholds at all octave frequencies tested hereafter referred to as all frequency average (AFA). This method of averaging was for a relatively better reflection of average hearing level. Based on AFA, the patients were grouped as per the three frequency average classification [29] as normal hearing (− 10 to 25 dB), mild (26–40 dB), moderate (41–55 dB), moderately severe (56–70 dB), severe (71–90 dB) and profound (> 90 dB) hearing loss. Pure tone audiometric findings were analyzed in terms of percentage of ears affected.

## 4 Statistical analysis

Descriptive statistics mean ± standard deviation (range) for continuous variables and number (percentage) for categorical variables were used to express data. All the data analyses were carried out using SPSS Ver. 13.0.

## 5 Results

Nine of the ten patients hailed from Southern India. There were seven males. The diagnosis was Familial MMND in 6 patients [FMMD variant-5, FMMND-1], Sporadic MMND in 4 cases [SMMND variant-2, SMMND-2]. Mean age at onset was 14.8 ± 4.34 years (range, 9–20). Mean age at presentation was 21.8 ± 6.5 years (range, 11–31). Mean duration of illness was 85.0 ± 54.7 months (3–168). Classically majority had a slender habitus. The salient neurological symptoms and signs among these 10 patients are shown in Table 1. The predominant initial symptom at onset was impaired hearing in 6 patients (60.0%). The neurological deficits were bilateral optic atrophy in 6 patients (60.0%) and associated mild visual impairment in 4 patients (66.6%). Bifacial weakness was noted in 5 patients (50.0%). Clinical hearing impairment in 10 patients (100.0%). Bulbar weakness in 10 patients (100.0%) [dysarthria in all 9 patients (90.0%), hypophonia in 9 patients (90.0%)]. Moderate to severe atrophy of the tongue with fasciculations was seen in 8 patients (80.0%). Wasting and weakness of distal muscles of the upper limbs were observed in 7 patients (70.0%). Wasting and weakness of distal muscles of the lower limbs were present in 3 patients (30.0%). Small muscles of the hand were severely weak when the upper limbs were affected. Signs of pyramidal tract dysfunction in the upper limbs were seen in 6 patients (60.0%), and in the lower limbs in 10 patients (100.0%). Babinski's sign was positive in 5 patients (50.0%). Mild cerebellar signs such as gait ataxia were found in 3 patients (30.0%). Higher mental functions were normal in all.

## 6 Audiological findings

Among the 20 ears (10 patients), various degrees of hearing acuity (from mild to profound hearing loss) were demonstrated. Pure tone audiometric findings in them were, sensorineural (SN) hearing loss of mild degree in 1 patient's ears (10.0%), moderate in 2 patient's ears (20.0%), moderately severe in 2 patient's ears (20.0%), severe in 2 patient's ears (20.0%) and profound degree in 3 patient's ears (30.0%).

The results of the distortion product (DP) otoacoustic emissions (OAE) and auditory brain stem response (ABR) were available for the 20 ears. The most significant finding was the presence of robust OAEs in the presence of impaired AFA (ranging from mild to profound degree of SN hearing loss) in all. Thus, DPOAE and ABR demonstrated a neural dysfunction.

## 7 Genetic findings

Screening of theSLC52A1, SLC52A2 and SLC52A3 genes in MMND cases and family probands by Sanger sequencing of the coding and flanking intronic regions did not reveal any pathogenic mutation. These genes were also analyzed using liquid based exome enrichment and sequencing and were found to also be negative. The C9ORF72 expansion was analyzed and found that the GGGGCC repeats were in the normal range of than 10.

## 8 Discussion

In this present report we describe a series of MMND patients and families. All 10 patients had the classical features of MMND/MMNDV with progressive hearing impairment associated with progressive involvement of bulbar nuclei (7th and 9th to 10th) including severe muscle weakness and wasting due to anterior horn cell involvement, with pyramidal dysfunction.

It is long been debated that MMND resembles Brown–Vialetto–Van Laere (BVVL) syndrome and the other complex childhood motor neuron disease syndromes which consist of Boltshauser syndrome, Nathalie syndrome and Fazio–Londe syndrome [24,30]. Over the last 110 years after its initial description, only 39 cases of BVVL syndrome have been documented worldwide [31]. In contrast we have seen more than 120 cases of MMND at a single neurological center over a 38 year period. BVVL syndrome is distinctly a rare familial disorder with slow or rapid progression of bilateral nerve deafness, involvement of the seventh to twelfth cranial nerves and rarely the third, fifth and sixth cranial nerves. Lower motor neuron signs in the limbs are infrequently present and pyramidal signs are rare [32], whereas in MMND lower and upper motor neuron signs are seen in majority of patients and third or sixth cranial nerve is never noted to be affected. In BVVL syndrome a female preponderance (M:F; 1:5) has been reported while in MNND a male preponderance or equal distribution is noted. In BVVL syndrome, more than 50% of reported cases are familial, and also autosomal dominant inheritance with variable penetrance and X-linked inheritance was postulated in some families [33].

This clinico-genetic study of MMND presents the clinical phenotype of a number of cases that have been characterized in detail. The genetic exclusion of the three genes that have been reported to cause the classical BVVL phenotype and the C9ORF72 which is the commonest cause of MND is important as it indicates that MMND is genetically distinct from these other forms of the disease. This suggests that MMND represents a distinct form of childhood onset motor neuron disease and does not, as previously suggested share any of the genes associated with BVVL like disorders.

The similarity of MMND with BVVL and the other early onset childhood motor neuron diseases suggests that the familial form MMND is likely to share a common or associated pathological pathway. It is also possible that MMND could be caused by a combination of genetic and environmental factors and of course we cannot exclude the possibility that these diseases could be associated with an unknown expanded repeat, as in the case of C9ORF72 which would be very difficult to identify using Sanger or next generation techniques. MMND is familial and sporadic and we suggest further analysis of the genetic and environmental factors to identify the causes of this disorder.

## Conflict of interest

The authors have no conflict of interest.

## Figures and Tables

**Table 1:** Clinical features of 10 patients with MMND. Where percentage is not applicable the expression is indicated beside the variable. SD = standard deviation, symm = symmetrical, asymm = asymmetrical, dis = distal, prox = proximal, UL = upper limbs, bil = bilateral.

Clinical characteristics	Total (n = 10)	
	Number of patients	Percentage
Age at presentation (mean ± SD) years (range)	21.8 ± 6.54(11–31)	
Age at onset (mean ± SD) years (range)	14.8 ± 4.34(9–20)	
Duration of illness (mean ± SD) months (range)	85.0 ± 54.7 (3–168)	
Symptom at onset		
Hearing impairment	6	60.0
UL wasting/weakness	2	20.0
Reduced vision	1	10.0
Hearing + vision	1	10.0
Neurological deficits		
Bilateral optic atrophy	6	60.0
Bilateral visual impairment	4	40.0
Bifacial weakness	5	50.0
Sensorineural deafness	10	100.0
IX to XII cranial nerves	10	100.0
Severe hypophonia	9	90.0
Atrophy and weakness		
Upper limbs	7	70.0
Symmetrical/asymmetrical	5/2	
Distribution		
Distal	7	100.0
Prox	3	42.8
Lower limbs	3	30.0
Symm/asymm	2/1	
Distribution		
Distal	3	100.0
Prox	0	
Deep tendon reflexes		
Upper limbs		
Normal	2	20.0
Sluggish/absent	2	20.0
Brisk	2	20.0
Exaggerated	4	40.0
Lower limbs		
Brisk	1	10.0
Exaggerated	9	90.0
Babinski sign	5	50.0
Cerebellar signs	3	30.0

**Table 2:** Primers used in the analysis childhood motor neuron disorders.

C20orf54	
C20orf54	Exon 2-1
Forward	TCACAGGAAGGGGAGTAATAAG
Reverse	CCAGCACCCAGGAGGTC
C20orf54	Exon 2-2
Forward	TCCGAAGTGCCCATCATC
Reverse	AGAAGGATGGAGGTGAGCAG
C20orf54	Exon 3-1
Forward	GCAGTCATTATTGCCACCTTG
Reverse	AGGCCACCAGGGTATAGAT
C20orf54	Exon 3-2
Forward	ACCAGGTCACCCTCCACTC
Reverse	TAGGTGCGTTTGGAATTCTG
C20orf54	Exon 4
Forward	TATGGAGACACTGGCCATCC
Reverse	CCCAAGCTCTCCCAGGC
C20orf54	Exon 5
Forward	GCCCTGTGAGAGTTCTTTGC
Reverse	GGCACTTGCGTTCATGATTC
GPR172A	
GPR172A	Exon 2
Forward	CAGTTCCCCTGGTCTCACC
Reverse	CACCCTCTGGAAGCTCTCTG
GPR172A	Exon 3-1
Forward	GCAGGTGTGCCCAAGACT
Reverse	GAAAACGCTCAAGGAAGTCG
GPR172A	Exon 3-2
Forward	ATGCTGTGCCTCGAATGTC
Reverse	GCTCTTGCAGTGGTGAGGAC
GPR172A	Exon 3-3
Forward	CCACCACCATCTGTACCCAC
Reverse	GAGCGAGCAGAATGTCAGG
GPR172A	Exon 4
Forward	GCTTTTCCTGCTTACCCTACG
Reverse	GAGAACACGCCAAGACACAG
GPR172A	Exon 5
Forward	GTGGTCCTCGTGGTGAGC
Reverse	CAGGCACTCAGGCATGG
GPR172B	
GPR172B	Primer pair 1
Forward	AGCATCTTTGGACCTACC
Reverse	TAGGAAGGCCACAGAGTG
GPR172B	Primer pair 2
Forward	GCCTGTGGTGGTAAAAGACC
Reverse	TAGGGCACTGAGACCCTGAC
GPR172B	Primer pair 3
Forward	CTGAGTGTAGTGGGCACAG
Reverse	ACCATGGGCTGAGAACAG
GPR172B	Primer pair 4
Forward	AGGAAGAAGAGGCTTTGC
Reverse	ACACAGACACAGCACCCAC
GPR172B	Primer pair 5
Forward	GAGCAAGTGGAGACATGAAG
Reverse	AGCCTCACGATGAAGACAG
